# Disentangling the effects of jasmonate and tissue loss on the sex allocation of an annual plant

**DOI:** 10.3389/fpls.2022.812558

**Published:** 2022-09-02

**Authors:** Nora Villamil, Benoit Sommervogel, John R. Pannell

**Affiliations:** Department of Ecology and Evolution, Université de Lausanne, Lausanne, Switzerland

**Keywords:** herbivory, defoliation, jasmonate, anti-herbivore defenses, hormone, sex allocation, sexual system, *Mercurialis annua*

## Abstract

Selection through pollinators plays a major role in the evolution of reproductive traits. However, herbivory can also induce changes in plant sexual expression and sexual systems, potentially influencing conditions governing transitions between sexual systems. Previous work has shown that herbivory has a strong effect on sex allocation in the wind-pollinated annual plant *Mercurialis annua*, likely *via* responses to resource loss. It is also known that many plants respond to herbivory by inducing signaling, and endogenous responses to it, *via* the plant hormone jasmonate. Here, we attempt to uncouple the effects of herbivory on sex allocation in *M. annua* through resource limitation (tissue loss) versus plant responses to jasmonate hormone signaling. We used a two-factorial experiment with four treatment combinations: control, herbivory (25% chronic tissue loss), jasmonate, and combined herbivory and jasmonate. We estimated the effects of tissue loss and defense-inducing hormones on reproductive allocation, male reproductive effort, and sex allocation. Tissue loss caused plants to reduce their male reproductive effort, resulting in changes in total sex allocation. However, application of jasmonate after herbivory reversed its effect on male investment. Our results show that herbivory has consequences on plant sex expression and sex allocation, and that defense-related hormones such as jasmonate can buffer the impacts. We discuss the physiological mechanisms that might underpin the effects of herbivory on sex allocation, and their potential implications for the evolution of plant sexual systems.

## Introduction

Flowering plants are remarkable for the diversity of their reproductive structures (flowers and inflorescences) and their sexual systems, which range from simultaneous hermaphroditism to completely separate sexes (dioecy), including intermediate systems in which males or females co-exist with hermaphrodites (Barrett, 2002). Much of this diversity is due to genetic differences that have accumulated between populations and species, but plant sexual systems are also often modulated by plastic responses of individuals’ sex expression to environmental or status-related cues, i.e., they reflect norms of reaction (Barrett, 2002; López and Domínguez, 2003; Cossard and Pannell, 2019). Pollinator-related selection is thought to be a major agent shaping both fixed and plastic aspects of plant sexual systems, but the influence of antagonists has probably been underestimated (Strauss et al., 1996; Carr and Eubanks, 2014; Johnson et al., 2015; Lucas-Barbosa, 2016; Santangelo et al., 2019). Herbivory, for instance, can affect floral display (Strauss et al., 1996; Santangelo et al., 2019), coloration (Strauss et al., 2004; Vaidya et al., 2018), floral scent (Kessler et al., 2011; Ramos and Schiestl, 2019), sex ratios (Hendrix and Trapp, 1981; Krupnick and Weis, 1998; Krupnick et al., 2000; Thomson et al., 2004) and plant sexual expression (Villamil et al., 2021). Moreover, selection on plant mating systems (e.g., through expression of self-compatibility/self-incompatibility) can influence the evolution of anti-herbivore defenses (Núnez-Farfán et al., 1996; Núñez-Farfán et al., 2007; Campbell and Kessler, 2013). Recent studies have exposed links between herbivory and aspects of plant mating, yet how this interaction affects the sex allocation in general and the expression of hermaphroditism versus dioecy specifically remains poorly understood.

Herbivory has at least two consequences for plants. Most directly, it causes tissue loss, reducing plant size and limiting resources available for maintenance, growth and reproduction through male and female sexual functions (Karban and Strauss, 1993; Lehtilä and Strauss, 1997, 1999; Mothershead and Marquis, 2000; Hambäck, 2001; Poveda et al., 2003; Ivey and Carr, 2005; Núñez-Farfán et al., 2007; Kessler et al., 2011). If herbivory influences fitness through male versus female allocation differently, then selection should favor a norm of reaction that we might label “conditional sex allocation” (Freeman et al., 1980; López and Domínguez, 2003; Hirata et al., 2019; Wang et al., 2021). Conditional sex allocation can imply complete sex changes when the fitness gains from the male or female sexual functions change with age or size (Ghiselin, 1969; Charnov, 1982), with selection favoring individuals that reproduce first through the sex whose reproductive value increases more slowly with size, and that then change to the other sex when they reach the age/size point at which fitness gains increase more rapidly (West, 2009). Although norms of reaction that entail complete switches in gender from male to female are known from some plants, e.g., jack-in-the-pulpit (Bierzychudek, 1984a,b; Policansky, 1987), changes in sex allocation are often quantitative in plants, with a more continuous shift in sex allocation from one sex function to the other (De Jong and Klinkhamer, 1989, 2005; De Jong et al., 1992; Klinkhamer et al., 1997; Pannell, 1997b; Pannell et al., 2008). Accordingly, reductions in plant size due to herbivory are most likely to elicit quantitative norms of reaction in sex allocation.

Herbivory also affects plants indirectly by triggering responses that help to prevent further attacks and to restore physiological equilibrium after damage. These responses can be endogenous, *via* electric, osmotic or hormonal signals within the plant (Salvador-Recatalà et al., 2014; Farmer et al., 2020), or exogenous, triggered by volatile signals from neighboring leaves/plants [e.g., jasmonate; (Heil and Bueno, 2007b; Heil, 2009; Ballaré, 2011)] or animals [e.g., herbivore eggs or saliva; (Hilker and Meiners, 2002; Mumm et al., 2003; Anastasaki et al., 2015)], which are perceived through leaf stomata. Signaling mediated by the volatile hormone jasmonate is particularly interesting because it is known to play a key role in both defense responses and the regulation of sex expression. On the one hand, jasmonate is released by damaged plant tissues and thus functions as an air-borne signal of nearby herbivore activity that induces anti-herbivore responses in leaves that perceive it (Thaler et al., 2001; Heil and Bueno, 2007b; Heil and Karban, 2010; Ballaré, 2011), either in undamaged leaves of the same plant, or in leaves from neighboring plants that “eavesdrop” on jasmonate signaling and induce their defenses in preparation for likely future damage (Heil and Bueno, 2007a,b; Karban, 2008; Karban et al., 2012). On the other hand, jasmonate is known to play a role in regulating sex expression, flower development, and sexual differentiation (Acosta et al., 2009; Yan et al., 2012; Wasternack et al., 2013; Cai et al., 2014; Yuan and Zhang, 2015) in a network of signals that may include cytokines, auxins and other hormones (Robert-Seilaniantz et al., 2011; Wasternack et al., 2013; Naseem et al., 2015; Yuan and Zhang, 2015). Importantly, however, the effects of jasmonate on the female or male components of sexual development vary among species (Wasternack et al., 2013). For instance, in *Arabidopsis thaliana* (Browse, 2009) and in maize (Yan et al., 2012), jasmonate is essential for male flower development, in tomato it is required for female flower development (Li et al., 2004), and in rice it determines the sex of the developing sexual organs (Acosta et al., 2009; Cai et al., 2014; Yuan and Zhang, 2015).

In this study, we explored norms of reaction in sex expression and sex allocation to herbivory in an experiment designed to uncouple its direct (through tissue loss) and indirect effects (due to defensive jasmonate signaling). Classic plant resource allocation theory suggests that increased investment in reproduction and growth should come at a cost in allocation to defense, and *vice versa* (Herms and Mattson, 1992). We thus tested the hypothesis that herbivory should cause plants to reduce their reproductive effort through a trade-off between resources allocated to defense versus reproduction. We reasoned that defense-related traits, such as those induced by jasmonate signaling, may link the coordinated evolution of reproductive and defensive traits. Growing evidence suggests that reproductive and defensive traits are not independent (Carr and Eubanks, 2014; Campbell, 2015; Johnson et al., 2015; Lucas-Barbosa, 2016), yet the effects of plant defensive strategies on trade-offs between male and female plant fitness and sex allocation still remain largely unexplored (Garcia and Eubanks, 2019).

To uncouple the direct and indirect effects of herbivory on sex expression, and to test the role of jasmonate on conditional sex allocation, we conducted a two-factorial experiment manipulating tissue loss (25% chronic defoliation) and plant anti-herbivore defenses *via* the jasmonate pathway (external application of jasmonate), and measured the sexual expression of plants with both a male and a female function. In many species (Reymond and Farmer, 1998; Han, 2016; Wang et al., 2019), plants that are damaged automatically release jasmonate into the environment, and it is therefore impossible for them to experience tissue loss without jasmonate release. However, our experiment included all other biologically possible combinations: un-manipulated control plants, plants subject to tissue loss only, plants subject to jasmonate only, and plants subject to tissue loss and jasmonate together.

Our experiment used females of the wind-pollinated annual dioecious plant *Mercurialis annua* that have evolved greatly enhanced “leaky” sex expression following the experimental removal of all males from their populations, as a result of selection for reproductive assurance and greater siring success (Cossard and Pannell, 2021; Cossard et al., 2021). Leaky sex expression (the production of gametes of the opposite sex by plants with separate sexes) is common in dioecious plants and likely plays an important role in reversions from dioecy to hermaphroditism (Ehlers and Bataillon, 2007; Cossard and Pannell, 2019). Because the genotypes used in our experiment are females that are now expressing a greatly enhanced leaky male-flower production that has evolved in the absence of herbivory, we had no *a priori* expectation for how they should respond to simulated herbivory. However, previous work on dioecious *M. annua* has shown that simulated herbivory enhances leaky sex expression in both males and females (Villamil et al., 2021), for reasons that may or may not be adaptive. We might thus have expected this tendency for greater leakiness under herbivory to have been retained or even enhanced in the genotypes we used in our experiment. Surprisingly, we found that females with leakiness enhanced through experimental evolution actually reduced their male flower production in response to tissue loss in our experiment, a response that was erased in plants that were exposed to hormone signaling by jasmonates. Our experiment thus reveals complex norms of reaction to herbivory that cannot be adaptive (because they are expressed by newly evolved genotypes), but that represent intrinsic pleiotropic responses to prior selection on reproductive allocation.

## Materials and methods

### Study system

*Mercurialis annua* (Euphorbiaceae) is an annual wind-pollinated herb distributed throughout central and western Europe and around the Mediterranean Basin (Tutin et al., 1968). The species has long been used as a model system to investigate the evolution of dioecy and sex expression (Yampolsky, 1919, 1930; Pannell, 1997b; Obbard et al., 2006) due to its great diversity and plasticity in sex expression and sexual systems (Yampolsky, 1930; Pannell, 1997a; Cossard and Pannell, 2021; Cossard et al., 2021). *M. annua* has chromosomal sex determination (XX♀; XY♂) (Russell and Pannell, 2015), mediated by endogenous hormonal signaling. Males are feminized by exogenous application of cytokinins, and females are masculinized by auxins (Durand and Durand, 1991). The plants used in this study are monoecious XX females that have recently evolved pollen production under natural selection under experimental evolution (Cossard et al., 2021) and are therefore now functionally hermaphroditic. These plants are an ideal system in which to test the effects of herbivory on sex allocation for two reasons. First, although *M. annua* has chromosomal sex determination, its sex expression is also hormonally regulated. Second, these plants have highly plastic sex allocation, which has evolved very recently; therefore, we expect the interference of defensive signals on sex allocation, if it occurs, to reflect deeply conserved physiological reaction norms that have not been shaped by recent natural selection. We are not aware of studies on the natural level of herbivory in wild populations of the diploid *M. annua*, but we know that in Western Europe its natural herbivores are slugs and snails (pers. obs.). Levels of natural herbivory are well-documented for the hexaploid populations of *M. annua*, which are androdioecious (populations in which males and hermaphrodites co-occur) and thus more similar in their sex allocation to the studied population. Herbivory is male-biased in the hexaploid *M. annua* populations, with males having damage levels two times greater than hermaphrodites (Sánchez-Vilas and Pannell, 2011), a consistent pattern amongst many plants with separate sexes (Cornelissen and Stiling, 2005).

### Plant culturing

Seeds generated through the previous selection experiment (Cossard et al., 2021) were sown in plastic trays with sterilized soil (Ricoter 163 soil) and kept under stable greenhouse conditions (25°C, 50% humidity; October 2019) at all times. When seedlings had flushed their first four leaves, approximately 3 weeks after sowing, they were transplanted into individual pots (Teku serie TO 14 D) with soil (Ricoter 140 soil) and slow-release fertilizer (Hauert Tardit: 3M 500 g for 100 L of soil). Plants were watered every 2 days by an automatic watering system.

### Experimental design

Pots were haphazardly assigned to one of four experimental treatments: control, herbivory only, jasmonate only, and herbivory and jasmonate together. For plants under the control treatment (C), leaves were sprayed with a sham solution containing only water and polysorbate until all leaves were wet (see Appendix for detailed solution formulae). The herbivory treatment (H) consisted of cutting off half of every second leaf on the plant with scissors and spraying plants with a sham solution until all leaves were wet (defoliation resulted in a 25% reduction of total leaf area over the course of the whole plant’s lifetime). Leaves were trimmed by cutting them in half perpendicularly to their midrib using scissors. In the jasmonate treatment (JA), plants were sprayed with a solution of methyl-jasmonate (concentration of 0.01%) and polysorbate until all leaves were wet (polysorbate 20 was used to fix the methyl-jasmonate on the sprayed leaves). Finally, the jasmonate and herbivory treatment (JAH) consisted of cutting off half of every other leaf on the plant with scissors and spraying plants with the methyl-jasmonate solution until all leaves were wet. These treatments were applied repeatedly as plants continued to grow, i.e., they represent chronic stress or manipulation. The first round of treatment was applied 1 week after repotting the plants (25th of November 2019) and then every 2 weeks over the next 12 weeks (the last treatment was applied on the 2nd of February 2020). On the first round of treatment, when most plants had fewer than six leaves each, we cut off only half a leaf (∼10% of the leaf area removed) for plants under the herbivory treatments to avoid seedlings death.

Because jasmonate is highly volatile and can be perceived through stomata by neighboring plants (Heil, 2009; Heil and Karban, 2010), plants from a given treatment were enclosed within two-meter curtains of transparent plastic to prevent “eavesdropping” cross-contamination. The eight identical enclosures consisted of an aluminum squared frame (130 cm × 130 cm) to which we attached four wooden poles (two meters long). The wooden structures were wrapped with 2 m-tall plastic curtains, which were fixed on three sides. The fourth side was held in place with pins acting as a door that allowed us to enter the enclosure for plant manipulations and measurements. To reduce the risk of contamination, enclosures remained closed at all times, and were opened only to allow access for measurements or manipulations. To avoid confounding effects of enclosure and treatment, we had a blocked design with two enclosures per treatment and 68 individuals per block.

### Sampling

Plant sampling consisted of cutting all above-ground plant material of 34 plants per enclosure (*N* = 272) and recording total height. Plants were then cut in half, lengthwise, creating two distinct segments: top and bottom. The top segment was carefully examined and we counted the number of fruits (immature and mature) and harvested all male flowers using tweezers. Male flowers were stored in paper envelopes, dried and weighed. After phenotyping, plant segments were dried and weighed to obtain plant dry biomass (top + bottom). To estimate seed production, the seeds were isolated from the dried plant materials, stored in paper envelopes and weighed. All materials were dried in an oven at 50°C for at least 14 days and weighed using a digital scale.

To increase sampling efficiency, we first conducted a pilot subsampling study to test whether flower production on the top half of the plant was correlated with the total flower production of the whole plant. A strong correlation would allow considering the top plant segments as a good estimate (hereafter, subsample) of the whole plant. Plant subsampling consisted of cutting all above-ground plant material of five plants per enclosure (*N* = 40) and recording total height. The plants were cut in half, lengthwise, creating two distinct segments: top and bottom, both of which were phenotyped following the procedure detailed above. The accuracy of the top segment as a valid subsample was tested using a correlation between reproductive structures on the top segment, *versus* reproductive structures on the whole plant (top + bottom segments). We found a significant and positive correlation (*r* = 0.71; *P* = 3.94^–07^) concluding that the top sections were an accurate representation of the whole plant flower production, and proceeded with phenotyping only the top segment of the remaining 232 plants. All further statistical analyses on reproductive effort were conducted considering only plant subsample data (top segment), even for those 40 plants which were entirely phenotyped.

### Statistical analyses

All statistical analyses were conducted in *R* version 4.03 (R Core Team, 2021). Mixed-effect models were fitted using the “lme4” package (Bates et al., 2015), residuals and model assumptions were checked using the “DHARMa” package (Hartig, 2022), and *post-hoc* Tukey comparisons were tested using the “multcomp” package (Hothorn et al., 2008), and variance explained by fixed and random factors was estimated using the “MuMIn” package (Bartón and Barton, 2018). Model specifications, estimates and statistics are reported in Table 1. We included days-post-treatment (DPT) in our statistical models to account for the effects that the period elapsed between the last treatment application and the plant sampling date may have on our response variables. For logistical reasons, our sampling was spread over 14 days by a team of six assistants. We included observer in our statistical analyses to account for possible differences among assistants.

**TABLE 1:** Model outputs on the effects of herbivory on male reproductive effort, female reproductive effort, total reproductive effort, and plant biomass of *Mercurialis annua*.

*Predictors*	Male reproductive effort		Female reproductive effort		Reproductive effort		Plant biomass	
	*Estimates*	*p*	*Estimates*	*p*	*Estimates*	*p*	*Estimates*	*p*
Treatment[C]	−5.740	**<0.001**	0.213	**<0.001**	0.226	**<0.001**	11.514	**<0.001**
	(−8.187, −3.293)		(0.157, 0.268)		(0.175, 0.277)		(7.983, 15.046)	
Treatment[H]	−1.153	**0.048**	0.031	**0.042**	0.020	0.083	−1.199	0.341
	(−2.295, −0.010)		(0.001, 0.060)		(−0.003, 0.043)		(−3.666, 1.268)	
Treatment[JA]	0.077	0.895	−0.009	0.559	0.009	0.433	0.186	0.882
	(−1.064, −1.218)		(−0.038, 0.021)		(−0.032, 0.014)		(−2.281, 2.654)	
Treatment[JAH]	−0.457	0.432	0.011	0.473	0.006	0.629	−0.461	0.714
	(−1.597, 0.684)		(−0.019, 0.040)		(−0.017, 0.029)		(−2.928, −2.006)	
DPT	−0.012	0.826	−0.004	**0.002**	−0.004	**0.001**	0.100	0.190
	(−0.0115, 0.092)		(−0.006, −0.001)		(−0.006, −0.001)		–(0.049, 0.250)	
Bottom biomass	0.094	0.062	−0.005	**<0.001**	−0.005	**0.002**		
	(−0.005, 0.194)		(−0.007, −0.003)		(−0.006, −0.001)			
**Random effects**								
σ^2^	7.36		0.00		0.00		15.97	
τ_00_	0.12_Box_		0.00_Box_		0.00_Box_		1.12_Box_	
	0.08_Observer_		0.00_Observer_		0.00_Observer_			
ICC	0.03				0.01		0.07	
N	8_Box_		8_Box_		8_Box_		8_Box_	
	6_Observer_		6_Observer_		6_Observer_			
Observations	273		273		259		273	
Marginal R^2^/Conditional R^2^	0.045/0.071		0.146/NA		0.109/0.118		0.022/0.086	

DPT, days-post-treatment; C, control; H, herbivory (25% chronic tissue loss); JA, jasmonate application; JAH, 25% chronic tissue loss and jasmonate application. Numbers in brackets show the upper and lower 95% CI of mean model estimates. Numbers in bold indicate significant differences (P < 0.05).

To test the effects of herbivory on the male (MRE) and female (FRE) reproductive effort, defined as the proportion of biomass allocated to each sexual function per gram of biomass of the top subsample, we used Gaussian mixed models. To deal with zeroes and meet normality assumptions, we transformed MRE data by adding a value ten times smaller than the smallest non-zero value within its range, and applied the following formula: log(*MRE*) = *log*(*MRE* + 1^−06^). Both of these models included as fixed effects treatment, DPT, and the biomass of the bottom plant section to account for the fact that larger plants invest more in reproduction; exclusion box (block) and observer were included as random effects.

We tested the effects of herbivory on plants’ investment in sexual reproduction using a Gaussian mixed model. Reproductive effort (RE), defined as the proportion of above-ground plant biomass allocated to sexual reproduction (including male and female flowers and fruits), was fitted as the response variable. Treatment, DPT, and the biomass of the bottom plant section were fitted as fixed effects; exclusion box (block) and observer were included as random effects. We tested whether plants responded to herbivory through compensatory growth using a Gaussian mixed model, fitting total above-ground plant biomass as the response variable, treatment and DPT as fixed effects, and the exclusion box (block) as a random effect (for this test, we did not include observer as a random effect because all samples were weighed by one person).

## Results

**FIGURE 1:**
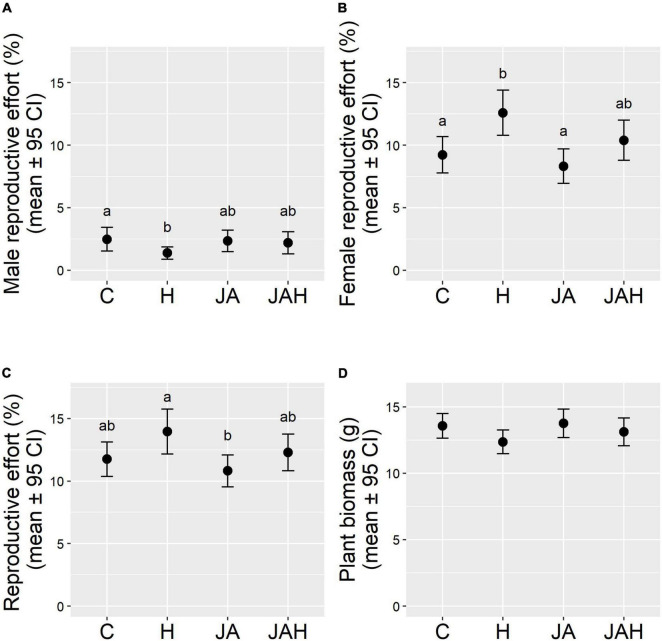
Direct (tissue loss) and indirect (jasmonate-mediated) effects of herbivory on **(A)** male reproductive effort (male inflorescence biomass/vegetative biomass), **(B)** female reproductive function (seed biomass/vegetative biomass), **(C)** total reproductive effort (reproductive biomass/vegetative biomass), **(D)** total aboveground plant biomass. Note that variables in panels **(A–C)** were calculated on the basis of measurements of the apical subsample for each individual sampled. Treatments are abbreviated as follows, C, control; H, herbivory treatment with a chronic tissue loss of 25% of foliar area; JA, jasmonate application; JAH, tissue loss and jasmonate application treatment. Different letters indicate significant differences between treatment levels (*P* < 0.05). Total *N* = 273 plants.

Overall investment in the male function represented less than 3% of total aboveground biomass in hermaphrodites, four times less than the proportion of plant biomass invested in the female function (Figures 1A,B). Tissue loss significantly halved male reproductive effort in plants that suffered herbivory compared to control plants (C: 2.47 ± 0.47; H: 1.36 ± 0.25; mean ± SE; Figure 1A), but jasmonate application restored male reproductive investment, as shown by the lack of significant differences between JAH and control plants (Figure 1A and Table 1). Interestingly, jasmonate alone did not enhance investment toward the male function, as shown by the non-significant differences between control plants and those only sprayed with jasmonate (Figure 1A and Table 1). The time elapsed between the applications of the last treatment and sampling date (DPT) did not significantly affect male reproductive effort (Table 1).

Tissue loss significantly increased female reproductive effort in hermaphrodites by a quarter compared to control plants or those only sprayed by jasmonate (C: 9.22 ± 0727; H: 12.58 ± 0.90; JA: 8.30 ± 0.69; mean ± SE; Figure 1B), but, again, jasmonate application in combination with tissue loss restored reproductive investment to levels similar to those of control plants, as shown by the lack of significant differences between JAH and control plants (Figure 1B and Table 1). The time elapsed between the application of the last treatment and sampling date (DPT) had a negative effect on female reproductive effort (Table 1). In all models, random effects explained very little of the variance of the response variables, indicating that the enclosure box and the observer had a negligible effect on our estimates (Table 1).

The herbivory treatments had no significant effect on plant biomass. Despite suffering 25% defoliation, plants under the herbivory treatment were on average only 8.85% lighter than control plants (C: 13.56 ± 0.46; H: 12.36 ± 0.44; mean ± SE; Figure 1D), a difference that was statistically non-significant (Figure 1D and Table 1). The time elapsed between the applications of the last treatment and sampling date (DPT) did not significantly affect plant biomass (Table 1). On average, approximately 25% of reproductive biomass was allocated to male flower production, whilst the remaining 75% was allocated toward the female function. However, neither tissue loss nor jasmonate application had a significant effect on the proportion of biomass allocated toward sexual reproduction (reproductive effort; Figure 1C and Table 1). Plants invested on average ∼10% of their total above-ground biomass toward reproduction, even under chronic 25% defoliation. Similarly, jasmonate application had no significant effect on reproductive effort (Figure 1C). The time elapsed between the application of the last treatment and sampling date (DPT) had a negative effect on reproductive effort (Table 1).

## Discussion

Even though chronic 25% defoliation had no significant effect on plant biomass, tissue loss significantly increased the proportion of biomass allocated toward reproduction. This increase in reproductive effort was invested toward the female function, at the expense of investment in the male function. However, exogenous jasmonate application effectively restored investment toward the male function in damaged plants.

### Herbivory-induced sex allocation was altered by selection in the absence of herbivory

Our experiment revealed that tissue loss due to chronic simulated herbivory in *M. annua* caused females with enhanced leaky sex expression in response to recent selection (Cossard et al., 2021) to shift their sex allocation away from male flower production. This response was thus opposed to that of their less leaky recent ancestors, for which tissue damage *increased* male flower production (Villamil et al., 2021). In other words, the very direction of the reaction norm of male flower production by females in response to tissue damage in natural populations of dioecious *M. annua* has been altered by strong selection on male flower production.

We do not know whether the reaction norm of male-flower production by females in the ancestral dioecious populations is adaptive, but it might be so (Villamil et al., 2021). However, we can safely say that the change in reaction norm that we have observed cannot be adaptive, for two reasons. First, females evolved enhanced male production in the selection experiment in the complete absence of sustained tissue damage (and more importantly in the absence of variation in tissue damage), so they were not exposed to selection that might have favored a different reaction norm. Second, the mean male flower production in the females of our experiment was much higher than is ever found in natural populations, such that, again, reaction norms around this new mean are not pertinent to the optimizing effects of natural selection in the ancestral population. The reaction norms we have measured are therefore clearly non-adaptive, and should properly be seen as a pleiotropic effect of selection on reproductive allocation.

Pleiotropic effects of natural selection have been found in selection experiments in a number of contexts (Hill and Caballero, 1992), including bacteria (Lenski, 1988a,b; Jasmin and Zeyl, 2013), insects (Harshman and Hoffmann, 2000), and plants (references cited below). A classic example is provided by Lenski’s *Escherichia coli* lines, which evolved resistance to a virus along with maladaptive mutations on various metabolic pathways and which eventually resulted in reduced fitness levels, similar to those in non-resistant populations (Lenski, 1988a,b). A more recent example in *Drosphila* shows that evolved resistance against an intestinal pathogen evolved along with deleterious effects on male fitness through reduced male mating success and reduced sperm competitive abilities (Kawecki, 2020). In plants, maladaptive pleiotropic effects in response to an experimental selection pressure have been widely observed in the context of evolution of herbicide resistance (Bergelson et al., 1996; Debban et al., 2015), but also in that of the evolution of flower color (Coberly and Rausher, 2008), induced defenses (Siemens and Mitchell-Olds, 1998), flowering phenology (Kover et al., 2009), or increased plant size leading to decreased defensive strategies and reduced floral signals (Zu and Schiestl, 2017). Our study provides a further revealing illustration of the complexity of multi-trait responses to selection, and, notably, of the fact that such responses include not only simple traits but also environmentally dependent reaction norms.

### Tissue loss caused plants to reduce male sex allocation, but jasmonate signaling restored it

Our experiment tested both the direct (tissue loss) and indirect (jasmonate signaling) effects of herbivory on sex allocation in the evolved population of *M. annua.* Interestingly, we found that plants reacted to both of these components differently. Male reproductive effort was nearly halved in defoliated plants, and jasmonate restored male investment after tissue loss, showing that signaling related to anti-herbivore defenses might also affect sexual expression. We expected the tissue loss and jasmonate application treatments to have additive effects on male investment, with the exogenous (jasmonate application) signal effectively amplifying the endogenous direct effects of tissue loss. However, jasmonate signaling caused plants to increase male net investment only if they had suffered tissue loss. To some extent, this result contrasts with previous findings showing that jasmonate can induce plant responses even in the absence of tissue loss (Heil et al., 2001a,b; Kost and Heil, 2008; Radhika et al., 2008; Kessler and Heil, 2011; Escalante-Pérez and Heil, 2012; Heil, 2015; Hernandez-Cumplido et al., 2016). If jasmonate on its own enhanced male allocation, whilst tissue loss reduced it, we expected plants that only received additional jasmonate but suffered no tissue loss (JA) to have greater male investment than those under the tissue loss and jasmonate treatment (JAH). However, we found no differences between plants in J and JAH treatments in male investment or any of the response variables measured. Our results thus show that an increased jasmonate signal perceived exogenously can restore male sex allocation only in damaged plants. As explained above, it is difficult to infer any functional or adaptive explanation for this result, but it is clear that different effects of damage interact and are not simply additive.

The exogenous application of jasmonate that restored male sex allocation to non-herbivory levels would be a favorably selected response in the recent evolutionary past of the *M. annua* population (in which males had been removed), because the male-flower production provides exceptionally high fitness through siring (Cossard and Pannell, 2021; Cossard et al., 2021). The ability of jasmonate to restore male sex allocation when plants are damaged may thus also be a pleiotropic effect of selection, perhaps as a result of a physiological constraint associated with hormonal “cross-talk.” Clearly, with time and appropriate conditions, we should expect natural selection to act upon the reaction norms we have observed, gradually modifying the non-adaptive response to jasmonate in restoring male allocation and converting it to a response that could be seen as adaptive. For instance, this could occur if plants evolved increased sensitivity to jasmonate as a male-function restorer so that the smaller amounts of jasmonate released exclusively by damaged plants would suffice to restore their male sex allocation in defoliated plants. Such an evolved response would allow damaged females with enhanced leakiness (H) to maximize their fitness, regardless of the herbivory levels experienced by their neighbors, i.e., the additional jasmonate levels. Future research in other species with a longer evolutionary history under monoecy and under suitable variation in herbivory levels would be valuable to determine whether jasmonate is also capable of restoring tissue loss-induced shifts in sex expression and what its adaptive or deleterious consequences might be.

Our findings suggest that jasmonate signaling may connect defensive and reproductive strategies in *M. annua*. More specifically, they indicate that herbivory might affect sex expression through its direct effects on tissue loss, but not through its indirect influence on hormone signaling, as perceived from neighboring plants (by eavesdropping), and that jasmonate perceived in synergy with damage might restore the effects of tissue loss. Jasmonate has been found to play a role in plant responses to herbivory (Thaler et al., 2001; Heil and Bueno, 2007b; Heil and Karban, 2010; Ballaré, 2011) and in regulating sex expression, flower development and sexual differentiation in a number of other plants species (Acosta et al., 2009; Yan et al., 2012; Wasternack et al., 2013; Cai et al., 2014; Yuan and Zhang, 2015), and our results confirm that it can act upon both functions simultaneously in *M. annua*; to our knowledge, this is the first account of the influence of jasmonate on the sexual expression of a species in the Euphorbiaceae family. *M. annua* has chromosomal sex determination (Durand and Durand, 1991; Russell and Pannell, 2015), but its sex expression can be influenced and even switched *via* the exogenous application of phytohormones such as cytokinins and auxins (Durand and Durand, 1991). The pleiotropic effects of herbivory on sex expression observed in *M. annua* could well be due to hormonal “cross-talk” between jasmonate and sex-determining hormones such as cytokinins and auxins, which has been well-documented in other species (Robert-Seilaniantz et al., 2011; Naseem et al., 2015).

### Tissue loss increased female sex allocation

Chronic defoliation elicited a change in male-flower production in *M. annua* females, and this change was associated with a corresponding response in fruit production, through a sex-allocation trade-off. This pattern contrasts with the available evidence on monoecious species in which defoliation had the opposite effect, i.e., reducing female, but not male allocation (Snyder, 1993; Quesada et al., 1995). Furthermore, our findings also contrast with previous studies in hermaphroditic species in which defoliation decreased male sex allocation, whilst female sex allocation remained unaffected (Allison, 1990; Frazee and Marquis, 1994; Lehtilä and Strauss, 1997). In this regard, our experimental population of monoecious *M. annua* plants seems to have an herbivory-induced sex allocation response different from those previously reported in the literature for hermaphroditic or monoecious species. As noted above, this unusual pattern may be attributable to the fact that it has not yet been molded by natural selection.

### No measurable negative impacts on performance and increased fitness: Evidence for tolerance?

Contrary to our expectations, tissue loss increased the proportion of biomass invested toward sexual reproduction. Despite a chronic 25% defoliation through the plants’ lifetime, we observed no trade-offs between growth and reproduction. Rather, we found that defoliated plants significantly increased their reproductive effort by 2% compared to control plants. Plants are known to respond to damage by herbivores in three main ways: by showing “resistance” (i.e., by investing in physical or chemical means to deter consumption); by “escaping” (i.e., evading attack through phenological mismatching); and/or through “tolerance” (i.e., compensating tissue loss with new growth) (Stamp, 2003; Boege et al., 2007; Fornoni, 2011). Tolerance can be considered the reaction norm of fitness in relation to a varying environment (i.e., damage), and although biomass is not a direct proxy of fitness, it can be related to fitness as a proxy of plant performance. Our experimental plants might have coped with damage through compensatory growth, given that we observed no measurable negative impacts of defoliation on performance (biomass) (Figure 1D). We conducted a power analysis using the *R* package “simr”; (Green and MacLeod, 2016) and found that, given our sample size (*N* = 272), our experiment had sufficient power (80% power) to detect effect sizes ≥ 25% on plant biomass, i.e., much greater than the effect sizes from our experiment (−8.5% and +1.3%). Thus, while it is possible that plants in our experiment compensated for lost tissue *via* a tolerance response, it lacked power to rule out substantial, non-compensatory, negative effects of herbivory.

### Concluding remarks

Our experimental design did not allow us to avoid the release of jasmonate by plants that suffered tissue loss, since this volatile is released with plant damage. Logistical constraints precluded treatments such as wrapping half of the leaves in foil to prevent their photosynthetic function (an equivalent of tissue loss) but without releasing jasmonate volatiles. Such an approach might be suitable for experiments in which the treatment was applied once or only a few times, but it was not possible to carry this out for a sample of >200 plants treated every 2 weeks. Adding a procedural control to test that the jasmonate induction treatment worked would also be worthwhile; indeed determining the jasmonate concentration required for successful inductions would provide interesting information for adjusting the dose required to induce plant defense at different ontogenetic stages.

Despite the above caveats, our results indicate that tissue loss and jasmonate signaling both had cascading effects on sex allocation in *M. annua*, and that jasmonate can be involved in both sex expression and defense pathways simultaneously. Our results thus suggest that defense-related traits—such as those induced by jasmonate signaling—may link the evolution of reproductive and defensive traits, and that jasmonate may thus play an important role in conditional sex allocation in some plants. Conditional sex allocation provides an ideal theoretical framework to address knowledge gaps on the effects of plant defenses on trade-offs between male and female plant fitness and sex allocation, advancing our understanding on the role antagonists may have as selective agents shaping sex allocation trade-offs. Appropriate experimental evolution studies would be a valuable tool to test the potential of selection to optimize this response.

## Data availability statement

The data presented in this study are deposited in the Zenodo repository: https://doi.org/10.5281/zenodo.5667348.

## Author contributions

NV conceived the ideas and designed the experiment. BS and NV collected and analyzed the data. NV and JP led the writing of the manuscript with critical inputs from BS. All authors contributed to the article and approved the submitted version.

## Appendix

### Appendix 1: Protocol for preparing sham and methyl-jasmonate solutions

Methyl-jasmonate solution:

1. 1 mL of Polysorbate 20 (tween20) gauged to 250 mL of distilled water.
2. 0.1 mL of Methyl-jasmonate gauged to 250 mL of distilled water.
3. Mix both solution, gauge to 1,000 mL and transfer to a jar with an airtight lid.
4. Vortex the mix 45 s (closed jar to avoid volatiles escaping) and keep the solution at 4°C.

Sham solution used for the control treatment.

1. 1 mL of Polysorbate 20 (tween20) gauged to 1,000 mL of distilled water.
2. Transfer to a jar with an airtight lid.
3. Vortex the mix 45 s (closed jar) and keep the solution at 4°C.

## References

[B1] Acosta I. F. Laparra H. Romero S. P. Schmelz E. Hamberg M. Mottinger J. P. (2009). tasselseed1 is a lipoxygenase affecting jasmonic acid signaling in sex determination of maize. *Science* 323 262–265. 10.1126/science.1164645 19131630

[B2] Allison T. D. (1990). The influence of deer browsing on the reproductive biology of Canada yew (*Taxus canadensis* marsh.). *Oecologia* 83 523–529. 10.1007/BF00317204 28313187

[B3] Anastasaki E. Balayannis G. Papanikolaou N. E. Michaelakis A. N. Milonas P. G. (2015). Oviposition induced volatiles in tomato plants. *Phytochem. Lett.* 13 262–266. 10.1016/j.phytol.2015.07.007

[B4] Ballaré C. L. (2011). Jasmonate-induced defenses: a tale of intelligence, collaborators and rascals. *Trends Plant Sci.* 16 249–257. 10.1016/j.tplants.2010.12.001 21216178

[B5] Barrett S. C. (2002). The evolution of plant sexual diversity. *Nat. Rev. Genet.* 3 274–284. 10.1038/nrg776 11967552

[B6] Bartón K. Barton M. (2018). *Package “MuMIn” – Multi-model Inference. R Package Version 1.42.1, Vienna, Austria.* Available online at: https://cran.r-project.org/web/packages/MuMIn/MuMIn.pdf

[B7] Bates D. Mächler M. Bolker B. Walker S. (2015). Fitting linear mixed-effects models using lme4. *J. Stat. Softw.* 67 1–48. 10.18637/jss.v067.i01

[B8] Bergelson J. Purrington C. B. Palm C. J. López-Gutiérrez J.-C. (1996). Costs of resistance: a test using transgenic *Arabidopsis thaliana*. *Proc. R. Soc. Lond. Ser. Biol. Sci.* 263 1659–1663. 10.1098/rspb.1996.0242 9025313

[B9] Bierzychudek P. (1984a). Assessing “optimal” life histories in a fluctuating environment: the evolution of sex-changing by jack-in-the-pulpit. *Am. Natural.* 123 829–840. 10.1086/284242

[B10] Bierzychudek P. (1984b). Determinants of gender in Jack-in-the-pulpit: the influence of plant size and reproductive history. *Oecologia* 65 14–18. 10.1007/BF00384456 28312103

[B11] Boege K. Dirzo R. Siemens D. Brown P. (2007). Ontogenetic switches from plant resistance to tolerance: minimizing costs with age? *Ecol. Lett.* 10 177–187. 10.1111/j.1461-0248.2006.01012.x 17305801

[B12] Browse J. (2009). The power of mutants for investigating jasmonate biosynthesis and signaling. *Phytochemistry* 70 1539–1546. 10.1016/j.phytochem.2009.08.004 19740496

[B13] Cai Q. Yuan Z. Chen M. Yin C. Luo Z. Zhao X. (2014). Jasmonic acid regulates spikelet development in rice. *Nat. Commun.* 5 1–13. 10.1038/ncomms4476 24647160

[B14] Campbell S. A. (2015). Ecological mechanisms for the coevolution of mating systems and defence. *New Phytol.* 205 1047–1053. 10.1111/nph.13212 25729803

[B15] Campbell S. Kessler A. (2013). Plant mating system transitions drive the macroevolution of defense strategies. *Proc. Natl. Acad. Sci. U.S.A.* 110 3973–3978. 10.1073/pnas.1213867110 23431190 PMC3593862

[B16] Carr D. E. Eubanks M. D. (2014). Interactions between insect herbivores and plant mating systems. *Annu. Rev. Entomol.* 59 185–203. 10.1146/annurev-ento-011613-162049 24160428

[B17] Charnov E. L. (1982). *The Theory of Sex Allocation.* Princeton, NJ: Princeton University Press.

[B18] Coberly L. C. Rausher M. D. (2008). Pleiotropic effects of an allele producing white flowers in *Ipomoea purpurea*. *Evolution* 62 1076–1085. 10.1111/j.1558-5646.2008.00355.x 18298642

[B19] Cornelissen T. Stiling P. J. O. (2005). Sex-biased herbivory: a meta-analysis of the effects of gender on plant-herbivore interactions. *Oikos* 111 488–500. 10.1111/j.1600-0706.2005.14075.x

[B20] Cossard G. G. Pannell J. R. (2019). A functional decomposition of sex inconstancy in the dioecious, colonizing plant *Mercurialis annua*. *Am. J. Bot.* 106 722–732. 10.1002/ajb2.1277 31081926

[B21] Cossard G. G. Pannell J. R. (2021). Enhanced leaky sex expression in response to pollen limitation in the dioecious plant *Mercurialis annua*. *J. Evol. Biol.* 34 416–422. 10.1111/jeb.13720 33098734 PMC7984330

[B22] Cossard G. G. Gerchen J. F. Li X. Cuenot Y. Pannell J. R. (2021). The rapid dissolution of dioecy by experimental evolution. *Curr. Biol.* 31 1277–1283. 10.1016/j.cub.2020.12.028 33472050

[B23] De Jong T. J. Waser N. M. Price M. V. Ring R. M. (1992). Plant size, geitonogamy and seed set in Ipomopsis aggregata. *Oecologia* 89 310–315. 10.1007/BF00317407 28313078

[B24] De Jong T. Klinkhamer P. (1989). Size-dependency of sex-allocation in hermaphroditic, monocarpic plants. *Funct. Ecol.* 3 201–206. 10.2307/2389301

[B25] De Jong T. Klinkhamer P. (2005). *Evolutionary Ecology of Plant Reproductive Strategies.* Cambridge: Cambridge University Press.

[B26] Debban C. L. Okum S. Pieper K. E. Wilson A. Baucom R. S. (2015). An examination of fitness costs of glyphosate resistance in the common morning glory, Ipomoea purpurea. *Ecol. Evol.* 5 5284–5294. 10.1002/ece3.1776 30151131 PMC6102511

[B27] Durand B. Durand R. (1991). Sex determination and reproductive organ differentiation in *Mercurialis*. *Plant Science* 80 49–65. 10.1016/0168-9452(91)90272-A

[B28] Ehlers B. K. Bataillon T. (2007). ‘Inconstant males’ and the maintenance of labile sex expression in subdioecious plants. *New Phytol.* 174 194–211. 10.1111/j.1469-8137.2007.01975.x 17335509

[B29] Escalante-Pérez M. Heil M. (2012). “Nectar secretion: its ecological context and physiological regulation,” in *Secretions and Exudates in Biological Systems*, eds Vivanco J. Baluška F. (Berlin: Springer-Verlag), 187–219. 10.1007/978-3-642-23047-9_9

[B30] Farmer E. E. Gao Y. Q. Lenzoni G. Wolfender J. L. Wu Q. (2020). Wound-and mechanostimulated electrical signals control hormone responses. *New Phytol.* 227 1037–1050. 10.1111/nph.16646 32392391

[B31] Fornoni J. (2011). Ecological and evolutionary implications of plant tolerance to herbivory. *Funct. Ecol.* 25 399–407. 10.1111/j.1365-2435.2010.01805.x

[B32] Frazee J. E. Marquis R. J. (1994). Environmental contribution to floral trait variation in *Chamaecrista fasciculata* (Fabaceae: Caesalpinoideae). *Am. J. Bot.* 81 206–215. 10.1002/j.1537-2197.1994.tb15431.x

[B33] Freeman D. Harper K. Charnov E. L. (1980). Sex change in plants: old and new observations and new hypotheses. *Oecologia* 47 222–232. 10.1007/BF00346825 28309476

[B34] Garcia L. C. Eubanks M. D. (2019). Overcompensation for insect herbivory: a review and meta-analysis of the evidence. *Ecology* 100:e02585. 10.1002/ecy.2585 30554427

[B35] Ghiselin M. T. (1969). The evolution of hermaphroditism among animals. *Q. Rev. Biol.* 44 189–208. 10.1086/406066 4901396

[B36] Green P. MacLeod C. J. (2016). SIMR: an R package for power analysis of generalized linear mixed models by simulation. *Methods Ecol. Evol.* 7 493–498. 10.1111/2041-210X.12504

[B37] Hambäck P. A. (2001). Direct and indirect effects of herbivory: feeding by spittlebugs affects pollinator visitation rates and seedset of *Rudbeckia hirta*. *Ecoscience* 8 45–50. 10.1080/11956860.2001.11682629

[B38] Han G.-Z. (2016). Evolution of jasmonate biosynthesis and signaling mechanisms. *J. Exp. Bot.* 68 1323–1331. 10.1093/jxb/erw470 28007954

[B39] Harshman L. G. Hoffmann A. A. (2000). Laboratory selection experiments using *Drosophila*: what do they really tell us? *Trends Ecol. Evol.* 15 32–36. 10.1016/S0169-5347(99)01756-5 10603505

[B40] Hartig F. (2022). *Package ‘DHARMa’. DHARMa: Residual Diagnostics for Hierarchical (multi-level/mixed) regression models. R Package Version 0.4.5, Vienna, Austria.* Available online at: https://CRAN.R-project.org/package=DHARMa

[B41] Heil M. (2009). “Airborne induction and priming of defenses,” in *Plant-Environment Interactions*, ed. Baluska F. (Berlin: Springer), 137–152. 10.1007/978-3-540-89230-4_8

[B42] Heil M. (2015). Extrafloral nectar at the plant-insect interface: a spotlight on chemical ecology, phenotypic plasticity, and food webs. *Annu. Rev. Entomol.* 60 213–232. 10.1146/annurev-ento-010814-020753 25564741

[B43] Heil M. Bueno J. C. S. (2007a). Herbivore-induced volatiles as rapid signals in systemic plant responses: how to quickly move the information? *Plant Signal. Behav.* 2 191–193. 10.4161/psb.2.3.4151 19704694 PMC2634055

[B44] Heil M. Bueno J. C. S. (2007b). Within-plant signaling by volatiles leads to induction and priming of an indirect plant defense in nature. *Proc. Natl. Acad. Sci. U.S.A.* 104 5467–5472. 10.1073/pnas.0610266104 17360371 PMC1838500

[B45] Heil M. Karban R. (2010). Explaining evolution of plant communication by airborne signals. *Trends Ecol. Evol.* 25 137–144. 10.1016/j.tree.2009.09.010 19837476

[B46] Heil M. Brigitte F. Ulrich M. Linsenmair K. E. (2001a). On benefits of indirect defence: short- and long-term studies of antiherbivore protection *via* mutualistic ants. *Oecologia* 126 395–403. 10.1007/s004420000532 28547454

[B47] Heil M. Koch T. Hilpert A. Fiala B. Boland W. Linsenmair K. E. (2001b). Extrafloral nectar production of the ant-associated plant, *Macaranga tanarius*, is an induced, indirect, defensive response elicited by jasmonic acid. *Proc. Natl. Acad. Sci. U.S.A.* 98 1083–1088. 10.1073/pnas.98.3.1083 11158598 PMC14712

[B48] Hendrix S. D. Trapp E. J. (1981). Plant-herbivore interactions: insect induced changes in host plant sex expression and fecundity. *Oecologia* 49 119–122. 10.1007/BF00376908 28309459

[B49] Herms D. A. Mattson W. J. (1992). The dilemma of plants: to grow or defend. *Q. Rev. Biol.* 67 283–335. 10.1086/417659

[B50] Hernandez-Cumplido J. Forter B. Moreira X. Heil M. Benrey B. (2016). Induced floral and extrafloral nectar production affect ant-pollinator interactions and plant fitness. *Biotropica* 48 342–348. 10.1111/btp.12283

[B51] Hilker M. Meiners T. (2002). “Induction of plant responses to oviposition and feeding by herbivorous arthropods: a comparison,” in *Proceedings of the 11th International Symposium on Insect-Plant Relationships*, (Dordrecht: Springer), 181–192. 10.1007/978-94-017-2776-1_21

[B52] Hill W. G. Caballero A. (1992). Artificial selection experiments. *Annu. Rev. Ecol. Syst.* 23 287–310. 10.1146/annurev.es.23.110192.001443

[B53] Hirata R. Wasaka N. Fujii A. Kato T. Sato H. J. P. E. (2019). Differences in flowering phenology, architecture, sexual expression and resource allocation between a heavily haired and a lightly haired nettle population: relationships with sika deer. *Plant Ecol.* 220 255–266. 10.1007/s11258-019-00910-7

[B54] Hothorn T. Bretz F. Westfall P. (2008). Simultaneous inference in general parametric models. *Biom. J.* 50 346–363. 10.1002/bimj.200810425 18481363

[B55] Ivey C. T. Carr D. E. (2005). Effects of herbivory and inbreeding on the pollinators and mating system of *Mimulus guttatus* (Phrymaceae). *Am. J. Bot.* 92 1641–1649. 10.3732/ajb.92.10.1641 21646081

[B56] Jasmin J.-N. Zeyl C. (2013). Evolution of pleiotropic costs in experimental populations. *J. Evol. Biol.* 26 1363–1369. 10.1111/jeb.12144 23638686

[B57] Johnson M. T. Campbell S. A. Barrett S. C. (2015). Evolutionary interactions between plant reproduction and defense against herbivores. *Annu. Rev. Ecol. Evol. Syst.* 46 191–213. 10.1146/annurev-ecolsys-112414-054215

[B58] Karban R. (2008). Plant behaviour and communication. *Ecol. Lett.* 11 727–739. 10.1111/j.1461-0248.2008.01183.x 18400016

[B59] Karban R. Strauss S. Y. (1993). Effects of herbivores on growth and reproduction of their perennial host *Erigeron glaucus*. *Ecology* 74 39–46. 10.2307/1939499

[B60] Karban R. Ishizaki S. Shiojiri K. (2012). Long-term demographic consequences of eavesdropping for sagebrush. *J. Ecol.* 100 932–938. 10.1111/j.1365-2745.2012.01974.x

[B61] Kawecki T. J. (2020). Sexual selection reveals a cost of pathogen resistance undetected in life-history assays. *Evolution* 74 338–348. 10.1111/evo.13895 31814118 PMC7028033

[B62] Kessler A. Heil M. (2011). The multiple faces of indirect defences and their agents of natural selection. *Funct. Ecol.* 25 348–357. 10.1111/j.1365-2435.2010.01818.x

[B63] Kessler A. Halitschke R. Poveda K. (2011). Herbivory-mediated pollinator limitation: negative impacts of induced volatiles on plant-pollinator interactions. *Ecology* 92 1769–1780. 10.1890/10-1945.1 21939073

[B64] Klinkhamer P. G. De Jong T. J. Metz H. (1997). Sex and size in cosexual plants. *Trends Ecol. Evol.* 12 260–265. 10.1016/S0169-5347(97)01078-1 21238063

[B65] Kost C. Heil M. (2008). The defensive role of volatile emission and extrafloral nectar secretion for lima bean in nature. *J. Chem. Ecol.* 34 1–13. 10.1007/s10886-007-9404-0 18071821 PMC2758370

[B66] Kover P. X. Rowntree J. K. Scarcelli N. Savriama Y. Eldridge T. Schaal B. A. (2009). Pleiotropic effects of environment-specific adaptation in *Arabidopsis thaliana*. *New Phytol.* 183 816–825. 10.1111/j.1469-8137.2009.02943.x 19594694

[B67] Krupnick G. A. Weis A. E. (1998). Floral herbivore effect on the sex expression of an andromonoecious plant, *Isomeris arborea* (Capparaceae). *Plant Ecol.* 134 151–162. 10.1023/A:1009762415520

[B68] Krupnick G. Avila G. Brown K. Stephenson A. (2000). Effects of herbivory on internal ethylene production and sex expression in *Cucurbita texana*. *Funct. Ecol.* 14 215–225. 10.1046/j.1365-2435.2000.00413.x

[B69] Lehtilä K. Strauss S. Y. (1997). Leaf damage by herbivores affects attractiveness to pollinators in wild radish, *Raphanus raphanistrum*. *Oecologia* 111 396–403. 10.1007/s004420050251 28308135

[B70] Lehtilä K. Strauss S. Y. (1999). Effects of foliar herbivory on male and female reproductive traits of wild radish Raphanus raphanistrum. *Ecology* 80 116–124. 10.1890/0012-9658(1999)080[0116:EOFHOM]2.0.CO;2 18707304

[B71] Lenski R. E. (1988a). Experimental studies of pleiotropy and epistais in *Escherichia coli* I. Variation in competitive fitness among mutants resistant to virus T4. *Evolution* 42 425–432. 10.1111/j.1558-5646.1988.tb04149.x 28564005

[B72] Lenski R. E. (1988b). Experimental studies of pleiotropy and epistasis in *Escherichia coli*. II. Compensation for maladaptive effects associated with resistance to virus T4. *Evolution* 42 433–440. 10.1111/j.1558-5646.1988.tb04150.x 28564011

[B73] Li L. Zhao Y. Mccaig B. C. Wingerd B. A. Wang J. Whalon M. E. (2004). The tomato homolog of CORONATINE-INSENSITIVE1 is required for the maternal control of seed maturation, jasmonate-signaled defense responses, and glandular trichome development. *Plant Cell* 16 126–143. 10.1105/tpc.017954 14688297 PMC301400

[B74] López S. Domínguez C. (2003). Sex choice in plants: facultative adjustment of the sex ratio in the perennial herb *Begonia gracilis*. *J. Evol. Biol.* 16 1177–1185. 10.1046/j.1420-9101.2003.00622.x 14640409

[B75] Lucas-Barbosa D. (2016). Integrating studies on plant–pollinator and plant–herbivore interactions. *Trends Plant Sci.* 21 125–133. 10.1016/j.tplants.2015.10.013 26598297

[B76] Mothershead K. Marquis R. J. (2000). Fitness impacts of herbivory through indirect effects on plant-pollinator interactions in *Oenothera macrocarpa*. *Ecology* 81 30–40. 10.2307/177131

[B77] Mumm R. Schrank K. Wegener R. Schulz S. Hilker M. (2003). Chemical analysis of volatiles emitted by Pinus sylvestris after induction by insect oviposition. *J. Chem. Ecol.* 29 1235–1252. 10.1023/A:1023841909199 12857033

[B78] Naseem M. Kaltdorf M. Dandekar T. (2015). The nexus between growth and defence signalling: auxin and cytokinin modulate plant immune response pathways. *J. Exp. Bot.* 66 4885–4896. 10.1093/jxb/erv297 26109575

[B79] Núnez-Farfán J. Cabrales-Vargas R. A. Dirzo R. (1996). Mating system consequences on resistance to herbivory and life history traits in *Datura stramonium*. *Am. J. Bot.* 83 1041–1049. 10.1002/j.1537-2197.1996.tb12801.x

[B80] Núñez-Farfán J. Fornoni J. Valverde P. L. (2007). The evolution of resistance and tolerance to herbivores. *Annu. Rev. Ecol. Evol. Syst.* 38 541–566. 10.1146/annurev.ecolsys.38.091206.095822

[B81] Obbard D. J. Harris S. A. Buggs R. J. Pannell J. R. (2006). Hybridization, polyploidy, and the evolution of sexual systems in *Mercurialis* (Euphorbiaceae). *Evolution* 60 1801–1815. 10.1111/j.0014-3820.2006.tb00524.x 17089965

[B82] Pannell J. (1997a). Mixed genetic and environmental sex determination in an androdioecious population of *Mercurialis annua*. *Heredity* 78 50–56. 10.1038/hdy.1997.6 16397637

[B83] Pannell J. (1997b). Variation in sex ratios and sex allocation in androdioecious *Mercurialis annua*. *J. Ecol.* 85 57–69. 10.2307/2960627 24618014

[B84] Pannell J. R. Dorken M. E. Pujol B. Berjano R. (2008). Gender variation and transitions between sexual systems in *Mercurialis annua* (Euphorbiaceae). *Int. J. Plant Sci.* 169 129–139. 10.1086/523360

[B85] Policansky D. (1987). Sex choice and reproductive costs in jack-in-the-pulpit. *Bioscience* 37 476–481. 10.2307/1310419

[B86] Poveda K. Steffan-Dewenter I. Scheu S. Tscharntke T. (2003). Effects of below-and above-ground herbivores on plant growth, flower visitation and seed set. *Oecologia* 135 601–605. 10.1007/s00442-003-1228-1 16228257

[B87] Quesada M. Bollman K. Stephenson A. G. (1995). Leaf damage decreases pollen production and hinders pollen performance in *Cucurbita texana*. *Ecology* 76 437–443. 10.2307/1941202

[B88] R Core Team (2021). *R: A Language and Environment for Statistical Computing.* Vienna: R Foundation for Statistical Computing.

[B89] Radhika V. Kost C. Bartram S. Heil M. Boland W. (2008). Testing the optimal defence hypothesis for two indirect defences: extrafloral nectar and volatile organic compounds. *Planta* 228 449–457. 10.1007/s00425-008-0749-6 18493790 PMC2459232

[B90] Ramos S. E. Schiestl F. (2019). Rapid plant evolution driven by the interaction of pollination and herbivory. *Science* 364 193–196. 10.1126/science.aav6962 30975889

[B91] Reymond P. Farmer E. E. (1998). Jasmonate and salicylate as global signals for defense gene expression. *Curr. Opin. Plant Biol.* 1 404–411. 10.1016/S1369-5266(98)80264-1 10066616

[B92] Robert-Seilaniantz A. Grant M. Jones J. D. G. (2011). Hormone crosstalk in plant disease and defense: more than just jasmonate-salicylate antagonism. *Annu. Rev. Phytopathol.* 49 317–343. 10.1146/annurev-phyto-073009-114447 21663438

[B93] Russell J. Pannell J. (2015). Sex determination in dioecious *Mercurialis annua* and its close diploid and polyploid relatives. *Heredity* 114 262–271. 10.1038/hdy.2014.95 25335556 PMC4815579

[B94] Salvador-Recatalà V. Tjallingii W. F. Farmer E. E. (2014). Real-time, *in vivo* intracellular recordings of caterpillar-induced depolarization waves in sieve elements using aphid electrodes. *New Phytol.* 203 674–684. 10.1111/nph.12807 24716546

[B95] Sánchez-Vilas J. Pannell J. R. (2011). Sex-differential herbivory in androdioecious *Mercurialis annua*. *PLoS One* 6:e22083. 10.1371/journal.pone.0022083 21779379 PMC3135621

[B96] Santangelo J. S. Thompson K. A. Johnson M. T. (2019). Herbivores and plant defences affect selection on plant reproductive traits more strongly than pollinators. *J. Evol. Biol.* 32 4–18. 10.1111/jeb.13392 30339305

[B97] Siemens D. H. Mitchell-Olds T. (1998). Evolution of pest-induced defenses in *Brassica* plants: tests of theory. *Ecology* 79 632–646. 10.1890/0012-9658(1998)079[0632:EOPIDI]2.0.CO;2

[B98] Snyder M. A. (1993). Interactions between Abert’s squirrel and ponderosa pine: the relationship between selective herbivory and host plant fitness. *Am. Nat.* 141 866–879. 10.1086/285513

[B99] Stamp N. (2003). Out of the quagmire of plant defense hypotheses. *Q. Rev. Biol.* 78 23–55. 10.1086/367580 12661508

[B100] Strauss S. Y. Conner J. K. Rush S. L. (1996). Foliar herbivory affects floral characters and plant attractiveness to pollinators: implications for male and female plant fitness. *Am. Nat.* 147 1098–1107. 10.1086/285896

[B101] Strauss S. Y. Irwin R. E. Lambrix V. M. (2004). Optimal defence theory and flower petal colour predict variation in the secondary chemistry of wild radish. *J. Ecol.* 92 132–141. 10.1111/j.1365-2745.2004.00843.x

[B102] Thaler J. S. Stout M. J. Karban R. Duffey S. S. (2001). Jasmonate-mediated induced plant resistance affects a community of herbivores. *Ecol. Entomol.* 26 312–324. 10.1046/j.1365-2311.2001.00324.x

[B103] Thomson V. Nicotra A. Cunningham S. (2004). Herbivory differentially affects male and female reproductive traits of *Cucumis sativus*. *Plant Biol.* 6 621–628. 10.1055/s-2004-821236 15375734

[B104] Tutin T. Heywood V. Burges N. Moore D. Valentine D. Walters S. (1968). *Flora Europaea, Vol. 2-5.* Cambridge: Cambridge University Press.

[B105] Vaidya P. Mcdurmon A. Mattoon E. Keefe M. Carley L. Lee C. R. (2018). Ecological causes and consequences of flower color polymorphism in a self-pollinating plant (*Boechera stricta*). *New Phytol.* 218 380–392. 29369384 10.1111/nph.14998

[B106] Villamil N. Li X. Seddon E. Pannell J. (2021). Herbivory enhances leaky sex expression in the dioecious herb *Mercurialis annua*. *Ann. Bot.* 129 79–86. 10.1093/aob/mcab129 34668537 PMC8829902

[B107] Wang J. Wu D. Wang Y. Xie D. (2019). Jasmonate action in plant defense against insects. *J. Exp. Bot.* 70 3391–3400. 10.1093/jxb/erz174 30976791

[B108] Wang Y. Luo A. Lyu T. Dimitrov D. Xu X. Freckleton R. P. (2021). Global distribution and evolutionary transitions of angiosperm sexual systems. *Ecol. Lett.* 24 1835–1847. 10.1111/ele.13815 34121305

[B109] Wasternack C. Forner S. Strnad M. Hause B. (2013). Jasmonates in flower and seed development. *Biochimie* 95 79–85. 10.1016/j.biochi.2012.06.005 22705387

[B110] West S. (2009). *Sex Allocation.* Princeton, NJ: Princeton University Press. 10.1515/9781400832019

[B111] Yampolsky C. (1919). Inheritance of sex in *Mercurialis annua*. *Am. J. Bot.* 6 410–442. 10.1002/j.1537-2197.1919.tb05554.x

[B112] Yampolsky C. (1930). Induced alteration of sex in the male plant of *Mercurialis annua*. *Bull. Torrey Bot. Club* 57 51–58. 10.2307/2480510

[B113] Yan Y. Christensen S. Isakeit T. Engelberth J. Meeley R. Hayward A. (2012). Disruption of OPR7 and OPR8 reveals the versatile functions of jasmonic acid in maize development and defense. *Plant Cell* 24 1420–1436. 10.1105/tpc.111.094151 22523204 PMC3398555

[B114] Yuan Z. Zhang D. (2015). Roles of jasmonate signalling in plant inflorescence and flower development. *Curr. Opin. Plant Biol.* 27 44–51. 10.1016/j.pbi.2015.05.024 26125498

[B115] Zu P. Schiestl F. P. (2017). The effects of becoming taller: direct and pleiotropic effects of artificial selection on plant height in *Brassica rapa*. *Plant J.* 89 1009–1019. 10.1111/tpj.13440 27889935

